# Blinded Prospective Evaluation of Computer-Based Mechanistic Schizophrenia Disease Model for Predicting Drug Response

**DOI:** 10.1371/journal.pone.0049732

**Published:** 2012-12-14

**Authors:** Hugo Geerts, Athan Spiros, Patrick Roberts, Roy Twyman, Larry Alphs, Anthony A. Grace

**Affiliations:** 1 In Silico Biosciences, Berwyn, Pennsylvania, United States of America; 2 Johnson & Johnson Pharmaceutical Research and Development, Central Nervous System Development, Titusville, New Jersey, United States of America; 3 Janssen Scientific Affairs, LLC, Titusville, New Jersey, United States of America; 4 Department of Neuroscience, Psychiatry, and Psychology, University of Pittsburgh, Pittsburgh, Pennsylvania, United States of America; Heidelberg University, Germany

## Abstract

The tremendous advances in understanding the neurobiological circuits involved in schizophrenia have not translated into more effective treatments. An alternative strategy is to use a recently published ‘Quantitative Systems Pharmacology’ computer-based mechanistic disease model of cortical/subcortical and striatal circuits based upon preclinical physiology, human pathology and pharmacology. The physiology of 27 relevant dopamine, serotonin, acetylcholine, norepinephrine, gamma-aminobutyric acid (GABA) and glutamate-mediated targets is calibrated using retrospective clinical data on 24 different antipsychotics. The model was challenged to predict quantitatively the clinical outcome in a blinded fashion of two experimental antipsychotic drugs; JNJ37822681, a highly selective low-affinity dopamine D_2_ antagonist and ocaperidone, a very high affinity dopamine D_2_ antagonist, using only pharmacology and human positron emission tomography (PET) imaging data. The model correctly predicted the lower performance of JNJ37822681 on the positive and negative syndrome scale (PANSS) total score and the higher extra-pyramidal symptom (EPS) liability compared to olanzapine and the relative performance of ocaperidone against olanzapine, but did not predict the absolute PANSS total score outcome and EPS liability for ocaperidone, possibly due to placebo responses and EPS assessment methods. Because of its virtual nature, this modeling approach can support central nervous system research and development by accounting for unique human drug properties, such as human metabolites, exposure, genotypes and off-target effects and can be a helpful tool for drug discovery and development.

## Introduction

Despite substantial research into the pathophysiology of schizophrenia, the current antipsychotic drugs based on dopamine (DA) D_2_ antagonism are not optimal in treating this disorder [1]. Although animal models have been invaluable in generating a better understanding of the schizophrenia pathophysiology and the mechanism of drug action, their inability to mimic the range of symptoms associated with this disorder [2] has hampered novel drug development.

In contrast, ‘Quantitative Systems Pharmacology’ is a novel approach based upon a computer-based biophysically realistic mechanistic disease model that can increase the parameter space beyond what can be informally and qualitatively be conceptualized. This approach uses extensive input from preclinical neurophysiology experiments and simulates a biophysically realistic model of the nucleus accumbens medium spiny neuron with a clinically determined striatal hyperdopaminergic tone [3] and cortical hypofrontality [4].

Drug effects are assessed by running their pharmacological profile against human receptors in a receptor competition model with neurotransmitter release based on realistic neuronal firing patterns that simulates receptor activation changes. In contrast to animal models, the computer-based model parameters are adjusted within biological ranges by optimizing the correlation of the model output calibrated using retrospective clinical outcomes of 24 antipsychotics at different doses.

This manuscript represents a unique collaboration among preclinical investigators, computer modelers and drug developers, and is highly innovative in that it utilizes basic drug pharmacology information and target engagement data of two novel antipsychotic agents to predict ***prospectively and blinded*** the actual clinical efficacy and extra-pyramidal symptoms (EPS) liability outcomes. An antagonist with a low affinity for D_2_ receptor, JNJ37822681 was developed based on the assumption that this would conserve its clinical efficacy with significantly lower EPS liability, similar to clozapine [5] and quetiapine [6]–[8], while the other compound, ocaperidone is a serotonin-dopamine antagonist with substantial off-target effects.

We will show in this report in a quantitative way that the lack of off-target effect especially at the 5-HT_2A_R will drive a substantial amount of EPS liability for the low-affinity, fast dissociating D2R antagonist JNJ37822681 that will result in a greater EPS liability than olanzapine at comparable D2R occupancies. Furthermore, the simulations will also suggest that the serotonin effect of ocaperidone will be unable to fully compensate for the same EPS liability.

This is, to the best of our knowledge, the first evaluation of the predictive validity of a computer model for the clinical efficacy and EPS liability, based solely upon the drug pharmacological profile and target engagement studies.

## Methods

**Figure 1:**
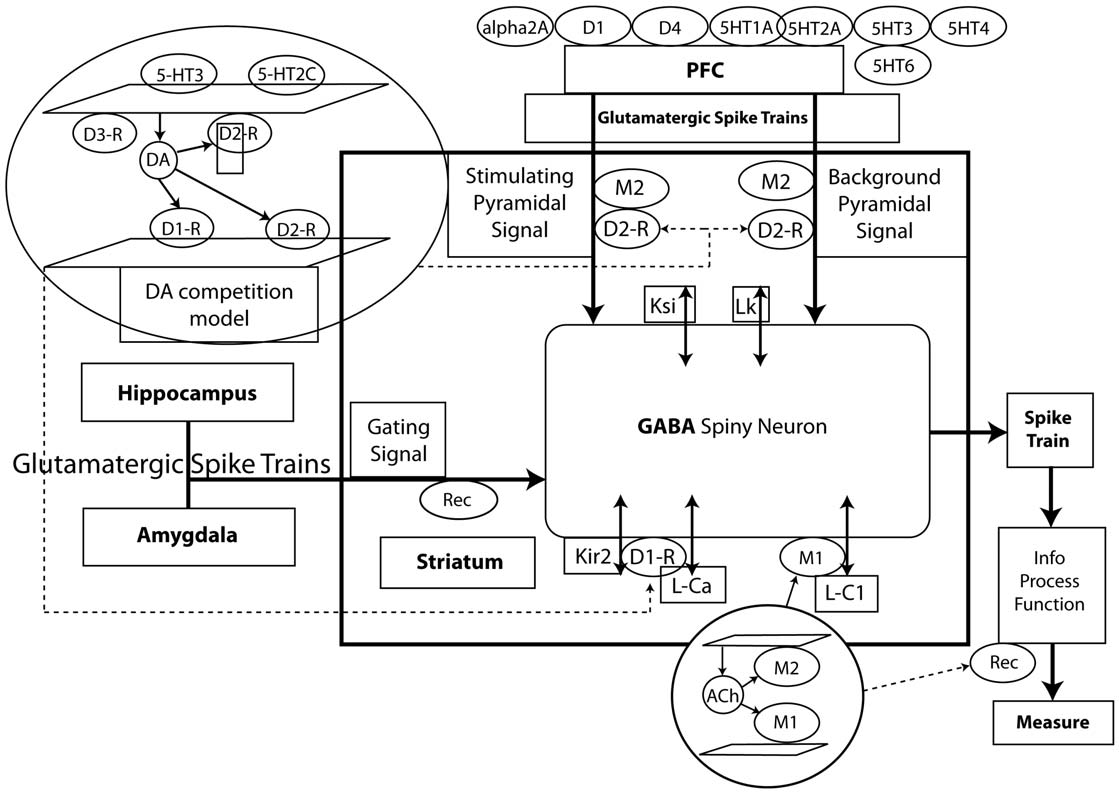
General overview of PANSS total computer-based model. This illustrates the PANSS total computer-based model that was based upon the neuroarchitecture and neurophysiology of the ventral striatum for a direct pathway medium spiny neuron (MSN), whose activation is driven by afferent projections from the cortex (further modulated by the D_2_ receptor), and background gating signals from hippocampus and amygdala and modulated by dopamine afferents from the ventral tegmental area through the D_1_ receptor. We combine this model with a similar model for the indirect pathway where postsynaptic D_2_ receptors modulate the excitability of the MSN. Serotonergic and noradrenergic tone is determined by dorsal raphe and locus coeruleus activity, while the cholinergic activity is derived from tonically active interneurons. Pharmacological agents can affect the model in a number of different ways [9]. We calculate the effect of the drug dose-combinations in the receptor competition model (as an example here the Dopamine DA receptor competition model), a set of differential equations that simulates the competition between neurotransmitter and active drug moiety under physiologically realistic conditions of presynaptic neuronal firing and autoreceptor coupling (see text for more details). Ten biological coupling parameters are fixed by optimizing the correlation outcome of this model for a large number of antipsychotic drug-dose combinations (43) and their reported clinical efficacy on PANSS total score (Rec = Receptor).

A more detailed description of the computer model is contained in an independent paper [9]. Briefly, a receptor competition model [10] simulates the competition between active moiety, tracer and neurotransmitter at relevant central synapses and yields accurate target exposure levels from imaging studies. A complex biophysically realistic subcortical nucleus (n.) accumbens model simulates the medium spiny neuron (MSN) dynamics with input from cortex, hippocampus and amygdala and modulation by 5-hydroxytryptamine (5-HT; serotonin), norepinephrine (NE) and acetylcholine (ACh) (Fig. 1). Finally a detailed computer model of a pyramidal cell in the supplemental motor area interacts with dorsal striatum MSN as components of the cortico-striatal-thalamo-cortical loop for the EPS model.

The model includes 27 relevant dopaminergic, serotonergic, cholinergic, adrenergic, glutamatergic and gamma-aminobutyric acid (GABA) receptors, implemented using preclinical data while the pathology is derived from human imaging and postmortem clinical data. The human pharmacology for each drug was determined from in vitro experiments performed at the Psychoactive Drug Screening Program (PDSP) and reported in the PDSP database (http://pdsp.med.unc.edu/indexR.html), where the affinity values are derived under the same standardized assay conditions. The reported active moiety pharmacology is combined with ^11^C-raclopride positron emission tomography (PET) imaging data to determine the functional target exposure of the different drug-dose combinations. With this drug concentration, the effect on postsynaptic receptor activation is calculated at all synapses using the appropriate pharmacological activity and the model output is calculated.

### B. Mathematical description of the PANSS subcortical n. accumbens module

The PANSS mathematical module simulates schizophrenia pathology and drug interventions on the action potential dynamics of a MSN in the n. accumbens, a key component of the circuitry involved in schizophrenia [11], [12]. Briefly, changes in MSN membrane potential are calculated using partial differential equations in NEURON [13], when driven by afferent cortical projections [14], gated by both hippocampal and amygdala projections (Fig. 1) and directly and indirectly modulated by dopaminergic, serotoninergic [15]–[16], cholinergic [17]–[19] and adrenergic [20] neurotransmitter systems [9].

We calculate the time-dependent changes in membrane potential V using Hodgkin-Huxley equations. For, instance, the inward rectifying potassium current, K_ir2_, is modified by the dopamine D_1_R activation u [21]–[22] so that the total current, 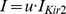. With a conductance, g_K_, and a reversal potential, E_K_ = −90 mV, the current takes on the form 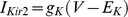 with a voltage dependent form
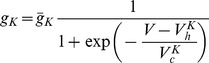
(1)where 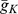 = 1.2 mS/cm^2^ is the maximum conductance, V_h_ = −111 mV is the value of the membrane potential that causes half activation and V_c_ = −11 mV describes the sensitivity of the change [23]–[24]. All simulations are coded in NEURON [25].

The model outcome is the number of action potentials over a predefined time period. Using another measure, based upon the interspike interval variability, essentially gives similar results. This model is repeated for a D_1_ MSN (for the direct pathway), a D_2_ MSN (indirect pathway) and a small percentage of D_1_+D_2_ containing MSN. D_1_R and D_2_R are coupled to different types of K^+^ channels on MSN, but both pathways do have a presynaptic D_2_R on glutamate neurotransmission onto MSN neurons [26]. The correlation between the individual models outcome already is high, but we combine them to be in line with the underlying striatal neurobiology. While many parameters are fixed from biological experiments, ten free biological coupling parameters (two for D_1_, D_2_, M_2_ and alpha_1_ and one for M_1_ and 5-HT_3_) are calibrated using the correlation between model outcome and the clinical readouts (43 drug-dose data points).

### C. EPS module

**Figure 2:**
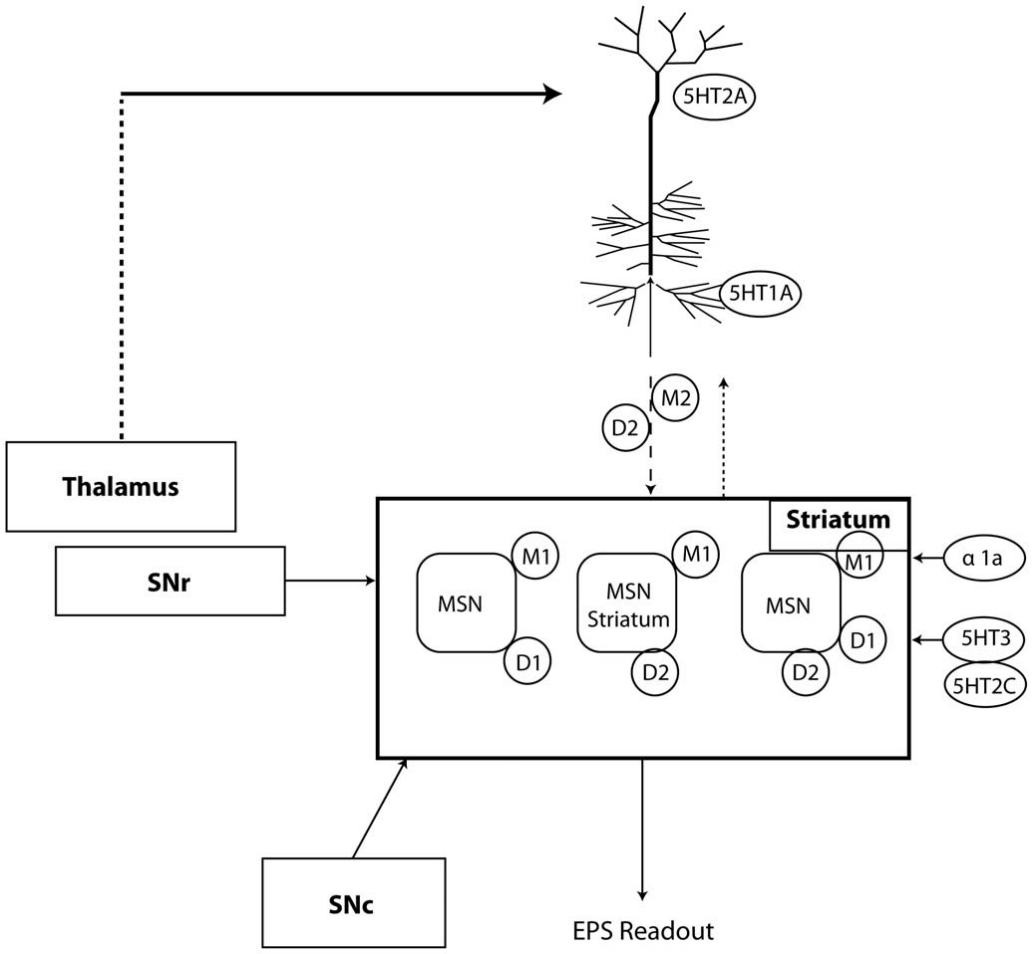
General overview of extra-pyramidal symptoms (EPS) computer-based model. This model was derived from the neuroarchitecture and neurophysiology of the relevant parts of the nigro-striatal motor pathway. We consider the D_1_-mediated direct pathway, the D_2_ mediated indirect pathway, and a pathway with both D_1_ and D_2_ receptors. We combine this with the effect of thalamic excitation on the supplementary motor area using a multi-compartment model of the pyramidal neuron [9]. Pharmacological agents can affect the model in many different ways. SN_r_ = substantia migra pars reticulata. SN_c_ = substantia nigra pars compacta.

The computer-based model for EPS (Fig. 2) has been described in detail previously [9] and consists of a biophysically realistic model for the dorsal striatum MSN based upon a direct D_1_ modulated pathway and an indirect D_2_ modulated pathway with a lower D_3_ autoreceptor level [27], in combination with a major input from the cortical supplemental motor area [28]. The MSN neuron model for the motor symptoms is very similar to the MSN model described above for the PANSS total model. The neuron in the cortical Supplementary Motor Area is modeled using a 12-compartment pyramidal cell with 5-HT_2_AR located at the apical dendrites [29] and 5-HT_1_AR [30] located in all compartments, and a threshold of input firing frequency on the apical dendrites is calculated that allows signals (calculated as membrane depolarization) to reach the cell soma. This rationale is based upon optogenetic studies in hemiparkinsonian mice that simulate robust D_2_R block by antipsychotics, suggesting that activity in the motor cortex is key for the pathological phenotype [31]. 5-HT_2_AR block and 5-HT_1_AR activation facilitate this process, thereby lowering the threshold through an effect on Na^+^ and Ca^2+^ [32] and on Ca ^2+^ channels respectively [33]. Based upon neuronal firing data from human deep brain stimulation [34], [35] in patients with and without Parkinsonian symptoms, we determined that the best EPS prediction would be given by multiplying the firing output from the motor MSN model with this threshold factor [9].

### D. Implementation of the schizophrenia pathology

Schizophrenia pathology is derived from in vivo imaging experiments and postmortem studies in schizophrenia patients, rather than exploring the causal relationship between different pathological processes [9]. For example, from human imaging studies that transiently deplete striatal dopamine [3], the amount of dopamine released in schizophrenics is about two-fold higher than in control subjects. Other changes include a decrease in D1R high affinity sites [36], a 30% decrease in D2R binding potential in chronically treated schizophrenia patients [37], unchanged D3R binding potential [27], a 30% decrease in DAT density [38] a decrease in 5-HT2CR expression [39], but no change in M1 and M2R expression [40].

The calibrated model related MSN firing to total PANSS score such that increased MSN firing over the fixed 21 second period leads to better total PANSS scores.

### E. The pharmacology of the tested compounds

Table 1 shows the pharmacology of the two test compounds as determined by in vitro affinity binding (data on file).

**Table 1:** Pharmacological affinities of the two experimental compounds (in nM) against human (JNJ37822681) or rodent receptors (ocaperidone).

Receptor	JNJ37822681	Ocaperidone	ND8295 (Ocaperidone metabolite)
D_1_	>10000	251	N/A
D_2_	220	1.22	1.3
D_3_	>10000	2.50	N/A
5-HT_1A_	>10000	17.17	19
5-HT_1B_	>10000	540	N/A
5-HT_1C_	>10000	28	N/A
5-HT_1D_	>10000	128.65	19
5-HT_2A_	1632	0.58	0.59
5-HT_2C_	>10000	27.00	32
5HT_3_	6692	750	N/A
5-HT_6_	5667	>10000	>10000
α_1_	>10000	0.46	0.66
α_2_	>10000	5.40	4.1
M_1_ mAChR	3000	1000	N/A
M_2_ mAChR	>10000	N/A	N/A
β_1_	>10000	750	N/A
β_2_	>10000	750	N/A
H_1_	2571	1.6	N/A
H_2_	>10000	500	N/A
Ca	3348	1500	N/A
GABA	>10000	1500	N/A

If there are no data available, we assume the compound does not affect that particular receptor. We further assume a 75∶25% distribution of the parent molecule ocaperidone and its metabolite ND8295 (data on file).

JNJ37822681 is a recently reported [41], [42] selective low-affinity D_2_ antagonist [43]–[45]. This compound is most similar to amisulpride with the major difference being that it is a low-affinity compound with a high off-rate, suggesting that it does not block the D_2_R during extended periods of dopamine release, as occurs during dopamine neuron burst firing.

Ocaperidone is a typical dopamine-serotonin antagonist with a high affinity for the D_2_ receptor (K_i_ 0.75 nM) [46] but with substantial off-target effects. This compound is similar to the atypical antipsychotics in that it affects a range of receptors at clinically relevant doses, including a substantial block of the 5-HT_2_AR.

For the missing pharmacology data we assume that the compound does not affect these receptors.

### F. Clinical trials

Briefly, the efficacy and safety of three fixed doses of JNJ37822681 administered twice daily in schizophrenics was studied in a randomized, double-blind, placebo- and active (olanzapine)-controlled, parallel-group study. For the purpose of the paper, the model prediction was compared for the five interventions against a PANSS total score, and EPS liability from spontaneous reporting of motor side-effects after 12 weeks (Clinicaltrials.gov identifier: NCT00728195).

The study population consisted of subjects with schizophrenia according to DSM-IV (295.10, 295.20, 295.30, 295.60, or 295.90) at least one year prior to screening and having experienced an acute exacerbation of less than six months duration, with a PANSS total score at baseline between 60 and 120 inclusive and aged 18 to 65 years (intent-to-treat [ITT] sample N = 99, 99, 103, 98, and 93, respectively, for placebo, 10, 20, 30 mg JNJ37822681, and 15 mg olanzapine). These doses resulted in raclopride displacement between 55 and 80% (see below) a range that included the average D2R occupancy of the comparator drug olanzapine. The existing antipsychotic medication had to be discontinued three to seven days before the first dosing with study medication. Although such a washout period may be perceived as somewhat short for some drugs, it is adequate for others. In the current environment washout periods of 3–7 days are considered ethically and are accepted by most clinical trial sites.

Ocaperidone was tested in a multi-center, double-blind placebo-controlled randomized parallel group dose titration study (N3D/FOROCA-05, data on file) in schizophrenia patients. Patients were included according to DSM IV-TR with PANSS total score >60 and a score >4 on any two of the P1, P3 and P6 subscales. Trial duration was six weeks and 0.6 mg active drug (n = 45) was tested vs. placebo (n = 43). This dose led to a D2 receptor target engagement of 69% (see below).

An older four week, double-blind, placebo-controlled trial (OCA-BEL4, data on file) with 2 to 20 mg haloperidol as active comparator used an average dose of 2.1 mg ocaperidone (n = 71) and 8.4 mg for haloperidol. Another 12-week double-blind active comparator-controlled trial (N3D/FOROCA-06, data on file) compared 0.40 mg ocaperidone (n = 53) to 15 mg olanzapine (n = 52).

## Results

### 1. Calibration of the mechanism-based computer model

This model is calibrated using publicly available clinical data on the PANSS total score collected in schizophrenia patients with stable medication that were switched to 24 different drugs and followed over maximal 12 weeks. As studies suggest that any clinical benefit is almost completely reached by the first 4 weeks [47], we collated all data on trials with durations between 4 and 12 weeks.

For each of the 71 drug-dose combinations the weighted average of the clinical outcome was calculated, with the number of patients in each individual group as the weighting parameter, resulting in a training set to adjust the ten coupling parameters for optimization. For the PANSS Total clinical scale we ended up with 43 different values for drug-dose combinations.

Functional human brain concentrations for each drug-dose combination were determined from the simulated displacement in the dopamine receptor competition model [10], where the active drug moiety competes with endogenous neurotransmitter and the tracer to reflect actual reported PET raclopride displacement in patients. Note that for calculating the effect of drugs on postsynaptic receptor activation levels, we used time-averaged values (10 seconds) of realistic in vivo firing frequencies as determined from both preclinical and where possible from human deep-brain stimulation data. For instance the dopaminergic striatal firing switches from tonic frequencies in the 1–4 Hz range to burst firing in the 40–80 Hz range [33]. Although this is a very dynamic process, only one average value is used to describe the effect of the drug on the receptor activation because the time scales of GPCR secondary pathways are likely much longer. The correlation coefficient between the PANSS total score clinical outcomes and the experimentally determined D_2_ receptor occupancies is modest (r^2^ = 0.18; p = 0.008) but in line with other reports [48], suggesting that functional D_2_ receptor occupancy only explains a small part of the variance with respect to the clinical outcome [9].

Introducing the same functional drug concentrations for the 43 drug-dose combinations in all the relevant synaptic models (DA, 5HT, NE, Ach, etc.) allows calculation of the drug effect on the change in postsynaptic receptor activations and subsequently on the disease model readout. These 43 outputs (number of spikes) were compared with the corresponding 43 clinical PANSS Total readouts. With the ten coupling parameters constrained biologically, we optimized the correlation using coarse grid searching in the 10-dimensional parameter space followed by the method of steepest descent with initial values determined by the coarse grid search. For example, for the optimal value of the coupling parameters 4 mg risperidone increases the firing number from 199 (placebo) to 245 for which the model predicts a PANSS total improvement of 13.4 points (11.8 measured). Similarly, 10 mg olanzapine corresponds to an MSN firing of 286 (PANSS total predicted 24.0 points vs. 22.7 points measured ), while a 210 mg dose of clozapine corresponds to an MSN firing number of 297 (PANSS Total predicted 27 points vs. 30 points measured).The full list of papers for the retrospective clinical database is available upon request to the authors.

This optimization resulted in a r^2^ = 0.59 between this outcome and the reported PANSS total score, much higher than the correlation (r^2^ = 0.18) between clinical outcome and D_2_ receptor occupancy [9], suggesting that the computer model correctly captures physiological off-target pharmacology beyond that predicted solely by D_2_ receptor occupancy. Similar correlation coefficients (r^2^ range between 0.27 and 0.73) were found with respect to other clinical efficacy readouts, such as the Brief Psychiatric Rating Scale (BPRS) and the Clinical Global Impressions Scale (CGI-S) and most are superior to the correlation coefficients found with the D_2_ receptor occupancy [9].

With all parameters fixed, the model was tested against different independent datasets. In one meta-analysis [49], the correlation coefficient between PANSS total score changes and the computer-based model was 0.20, compared to 0.09 between PANSS total score changes and D_2_ receptor occupancy. Another meta-analysis [50] studied 10 antipsychotic drugs at low and high doses; the correlation coefficient between PANSS total score changes and computer model outcome was 0.56 versus 0.11 for the correlation with the D_2_ receptor occupancy. The computer-based model also outperforms the D_2_ receptor occupancy correlation in the Clinical Antipsychotic Trials of Intervention Effectiveness (CATIE) dataset for five of the eight readouts [9] that probe real-life efficacy of different antipsychotics [1].

A sensitivity analysis around the calibrated parameters revealed a high sensitivity for D_2_>D_1_>5-HT_3_>alpha_1_>M_2_>M_1_ in descending order. For a positive 20% deviation from the calibrated value of D2 we observe a change between −2.2 and −5.2 points for the different drugs (average −3.12); conversely for a negative 20% deviation a range between 0.5 and 2.9 change in PANSS Total is observed (average 1.70).

As a comparison, we performed a multivariate correlation analysis between the receptor occupancies of each drug-dose combination and their respective PANSS Total outcomes. We restricted the receptors to those that had known physiology in the subcortical regions used in our PANSS model. The receptor occupancies were calculated using the formula Occ = Dose/(Dose+K_i_) where K_i_ is the dose at which half of the receptors are occupied. Because displacement data at all the receptors in the model are not known, in a first approximation, we set K_i_ = K_D2_ (Aff-_x_/Aff-_D2_) where K_D2_ is the dose at which raclopride displacement from the D2R is 50%. Aff-_x_ and Aff-_D2_ are the affinities of the drug for the receptor X (any of the non-D_2_R) and D_2_ respectively. Using this approximation, the correlation then increases from 0.19 for the single D2R occupancy to 0.35, still lower than the correlation we could achieve with our physiology-based model, despite having a similar number of degrees of freedom.

The EPS computer-based model was calibrated similarly using the fraction of patients with anticholinergic therapy as reported clinical EPS liabilities in the same patient population. The correlation between this clinical outcome and the D_2_ receptor occupancy (r^2^ = 0.03) [9] is much lower than the correlation between this outcome and the EPS computer-based model (r^2^ = 0.39) [9]. With these values, the threshold for cortical pyramidal firing for example decreases from 0.70 in the placebo case to 0.62 for the olanzapine 10 mg case. Using the multivariate regression approach mentioned above, we get a slightly better correlation of 0.49. A new therapeutic intervention can then be simulated in the model, based upon its pharmacology and functional brain concentration derived from target engagement studies leading to a clinical outcome prediction with a 95% prediction interval.

### 2. Calculation of the functional brain concentration for the two investigative compounds

The reported ^11^C-raclopride imaging studies for the two compounds as a function of the dose was used in the receptor competition model of the primatized dopaminergic synapse [10] with drug, dopamine and tracer – each with appropriate affinities – to determine the functional intrasynaptic concentrations that matched the observed tracer displacement. From the observed 55%, 75% and 80% D_2_ receptor occupancy for the three doses of JNJ37822681, we determined effective functional brain concentrations of 400, 700 and 840 nM, while 15 mg olanzapine corresponds to a D_2_ receptor occupancy of 75% [51]. A single dose-study with limited numbers of subjects matched the observed ^11^C raclopride displacement of 69% at 0.52 mg ocaperidone to a 4.3 nM concentration.

### 3. Prediction of the clinical PANSS total score and EPS outcome for the two compounds

**Figure 3:**
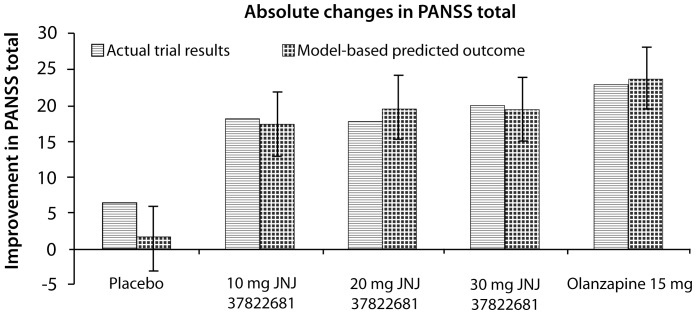
Predicted and actual clinical (PANSS) outcomes using the Computer-based model. This illustrates the predicted and actual clinical outcomes for placebo, three doses of JNJ37822681 and the comparator olanzapine on the difference in PANSS total score clinical scale between week 8 and the baseline. Except for the placebo effect, the computer-based model predicts the clinical outcome quite well, especially the relative performance of JNJ37822681 and the active comparator in terms of actual PANSS total scores. Error bars reflect the 95% prediction intervals derived from the predicted ANOVA analysis.

The pharmacology of the compounds (Table 1) was subsequently entered in the PANSS and the EPS computer models for each of the relevant doses and the predicted clinical scales were calculated from the model output using the correlation functions [9]. All clinical PANSS total score outcomes for JNJ37822681 were significantly different from placebo, but not from olanzapine (Fig. 3). The computer model (error bars = 95% Prediction Interval) accurately captures the relative order of the clinical PANSS total score outcome for all treatment arms with JNJ37822681 versus olanzapine, although the absolute change for the placebo arm is greatly underestimated (1.7 points in the model versus 6.4 measured). For instance, the model predicted an absolute PANSS total score change from baseline of 19.4 points (measured 20 points) for 30 mg JNJ37822681 and 23.7 points (measured 22.9); an effect for olanzapine that was inversely proportional to the D_2_ receptor occupancy (80% versus 75% for olanzapine). Along the same lines, the computer model predicts a −25.2 point change for 250 mg of the weak D_2_ receptor antagonist clozapine.

It is of interest to compare this predicted outcome with the clinical outcome predicted by the multivariate analysis above. Placebo value (all data are improvements from baseline) would be 3.4 (measured 6.4), outcome for 10 mg JNJ would be 16 (measured 18), for 20 mg JNJ (20.7 vs.. 17.7 measured), for the 30 mg JNJ 21.9 vs. 20.2 measured and for olanzapine 20.7 (measured 22.9). This analysis underestimates placebo and olanzapine and overestimates the JNJ effect.

**Figure 4:**
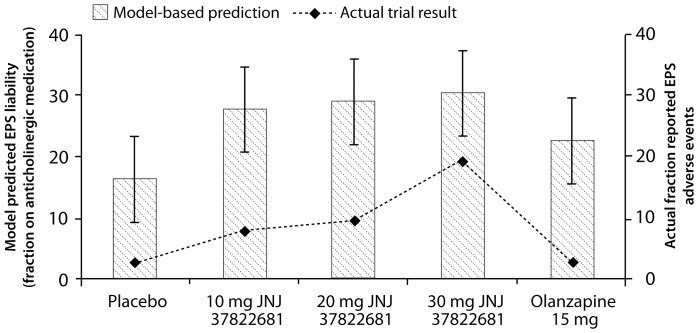
Predicted and actual clinical outcomes of JNJ37822681 and olanzapine on EPS liability. This figure illustrates the predicted and actual clinical outcomes of JNJ37822681 and the comparator olanzapine on EPS liability. Error bars reflect the 95% prediction intervals derived from the predicted ANOVA analysis. Note that the clinical readout (fraction of patients reporting EPS side-effects) and model readout (fraction of patients needing anticholinergics) are two different readouts of the same clinical effect. The computer-based model correctly identifies the greater and unexpected EPS liability of the two highest doses of JNJ37822681 compared to the olanzapine treatment, despite having a lower or identical D_2_ receptor occupancy.

Interestingly and unexpectedly, the computer-based model predicted a higher EPS liability for JNJ37822681 as compared to olanzapine (EPS reported liabilities 3%, 8%, 10%, 19% and 3% for placebo, 10, 20, 30 mg JNJ37822681 and olanzapine, respectively; model predicted values 16%, 27%, 28%, 29% and 23% for anticholinergic medication use). EPS clinical readouts are statistically significant for 20 and 30 mg JNJ37822681 versus placebo or olanzapine (Fig. 4). The EPS scale is different from the EPS scale used for calibration, resulting in different absolute outcomes; however, the computer model correctly predicted the relative risk for Parkinsonian-related side-effects for the therapeutic interventions. This result contrasts with the underlying rationale for this Research and Development project assuming that weak D_2_ receptor affinity combines good clinical efficacy with a lower EPS liability as observed in preclinical studies [42]. Also the computer model correctly captures the off-target olanzapine pharmacology that reduces EPS liability compared to the 10 mg JNJ37822681, despite a higher D_2_ receptor occupancy (75% versus 55%).

The multivariate regression model predicts an EPS liability of 11% for placebo, 34%, 43% and 45% for the three doses of JNJ37822681 versus 20% for olanzapine, also confirming the much greater EPS liability; however note the greater difference between placebo and olanzapine (almost a doubling in frequency versus an increase from 16 vs. 23% for the computer-based model).

**Figure 5:**
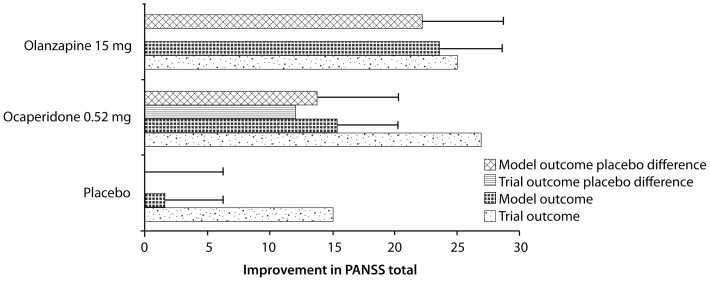
Predicted and actual clinical (PANSS) outcomes of ocaperidone and the comparator olanzapine. The panel illustrates the difference in PANSS total clinical scale between the end of the trial and the baseline (derived from three different trials). Error bars reflect the 95% prediction intervals derived from the predicted ANOVA analysis. There is a substantial placebo effect that the computer-based model cannot predict; however, the difference between placebo and treatment with ocaperidone is correctly accounted for.

For ocaperidone, the computer-based model wrongly predicted the absolute PANSS total score changes from baseline in the placebo (14.5 points) and ocaperidone (26.9 points), but better predicted the improvement of ocaperidone-treated patients with the placebo-difference subtracted (12.4 measured versus 13.7 for the computer-based model) (Fig. 5). Note that the multivariate analysis predicted a placebo difference subtraction effect of 9.6 points, much less than the 12.4 points measured. In the active comparator trial with olanzapine (N3D-OCA6) the computer-model clearly underestimated the clinical effect of ocaperidone, measured as a difference from baseline (15.1 predicted versus 23.1 measured), but better predicted the clinical effect of olanzapine (22.1 predicted versus 24.9 measured). Here the multivariate analysis predicted values of 13.05 for ocaperidone and 20.7 for olanzapine; which is in general worse than the computer-based model outcome.

**Figure 6:**
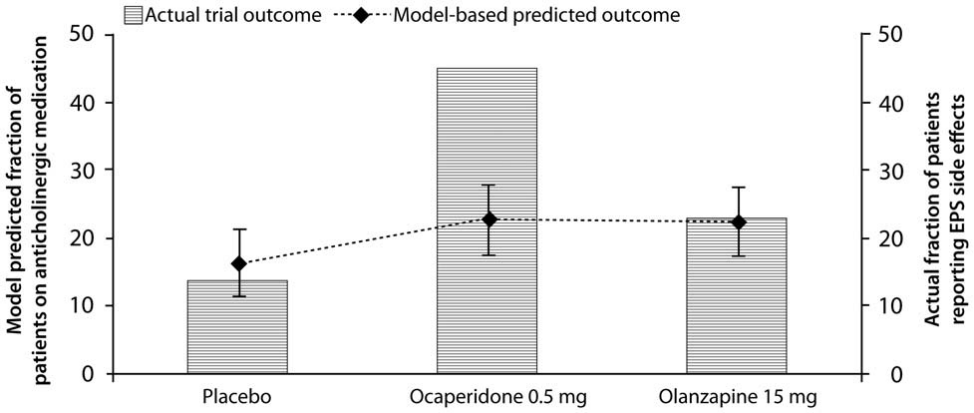
Model predicted and actual clinical outcomes of ocaperidone and olanzapine on EPS liability. This illustrates the model predicted and actual clinical outcomes of ocaperidone and the comparator olanzapine on the EPS liability. Error bars reflect the 95% prediction intervals derived from the predicted ANOVA analysis. While the computer-based model quite accurately described the EPS liability of 15 mg olanzapine, it greatly underestimated the EPS liability of ocaperidone. As described in the text, assuming higher receptor occupancy for ocaperidone substantially increases the EPS liability of ocaperidone.

In the active comparator trial N3D-OCA6, the computer-based model underestimated EPS liability for ocaperidone (23.5% predicted versus 44% measured) but correctly predicted olanzapine's EPS liability (23% predicted versus 23.5% measured) (Fig. 6). The multivariate regression analysis underestimates even more the EPS liability of ocaperidone and olanzapine (18% for ocaperidone and 20% for olanzapine).

We simulated the scenario that the reported single-dose ocaperidone imaging study underestimated the real target exposure. For 75, 85 and 90% D_2_ receptor occupancy respectively the PANSS total improvement in the model outcome increased to 19.1, 20.6 and 21.3 points, while the EPS liability increased from 22% to 28%, 31% and 32%. A higher actual ocaperidone target engagement could explain a substantial part of the divergences between model outcome and clinical outcome.

In contrast, using the multivariate regression model, the predicted PANSS Total for ocaperidone would increase to 13.8, 14.8 and 14.6 points on the PANSS Total Scale for these three conditions; certainly a much lower increase than for the computer-based model. With regard to the EPS outcome, the multivariate regression analysis would increase the EPS liability from 0.18 to 0.20, 0.23 and 0.24 respectively; much smaller increases than with the computer-based model outcome.

## Discussion

The optimal antipsychotic drug dose in patients is usually based on PET imaging with an optimal window between 70% and 80% of D_2_ receptor occupancy [52]–[54], with an exception for the partial D_2_ receptor agonist aripiprazole. However the D_2_ receptor occupancy only partly accounts for the clinical response and our increased understanding of other neurotransmitter systems and systems interactions has not been effectively integrated into antipsychotic drug discovery. We demonstrate here a quantitative mechanistic computer-based model as a translational tool that combines preclinical physiology data with patient-centered data on neuronal circuits, pathology and pharmacology, eliminating some of the inherent limitations of preclinical animal models [55]. Due to its mechanistic nature this model is limited to specific disease areas like schizophrenia, in contrast to the more generic systems biology data-mining approaches often applied to different disease areas.

We showed that retrospective evaluation of drug efficacy with a wide range of pharmacological activities using this computer model is more effective than simple receptor D_2_ receptor competition or multivariate regression analysis. We have further tested this translational model by predicting, in a blinded manner, the clinical profile of two compounds for which clinical data had been collected but not published or available to the modelers at the time of evaluation. To our knowledge, this is the first time that any simulation model has been tested in such a blinded manner.

The results suggest that the mechanistic disease model correctly predicts the relative performance for JNJ37822681 in PANSS total score improvement and EPS liability with respect to olanzapine, but not for ocaperidone. The low-affinity property of JNJ37822681 differentially modulates only the dopaminergic striatal pathway effects during burst and tonic dopamine activity. The model prediction of the potent clinical improvement with clozapine and of the efficacy of olanzapine as compared to the two highest doses of JNJ37822681, despite the same or lower D_2_ receptor occupancy also suggests that the computer model adequately captures the beneficial contribution of additional non-D_2_ receptor actions.

In line with the reported clinical benefit of trazodone in Parkinson disease patients [56], our model suggests that cortical 5-HT_2_A activity is a key modulator of EPS liability and that the fast dissociation rate of JNJ37822681 may only compensate partially for the EPS liability induced by significant D_2_ receptor inhibition during burst firing, This is not unlike remoxipride that has a substantial EPS liability despite a low affinity for the D_2_ receptor [57]. We believe this translational disconnect is likely due to species difference of the dopaminergic synapse physiology between primates and rodents [10].

While a simple multivariate regression analysis can already give a good idea on the expected outcomes, our analysis suggests that the mechanism-based computer model is superior in predicting the clinical outcomes of both JNJ37822681 and ocaperidone. This is likely due to the fact that the multivariate analysis assumes independent processes that affect the outcome in a linear way. In contrast actual physiological modeling can account for a non-linear processes such as the threshold for action potential generation or the complex non-linear interaction between different receptor systems (for instance one neurotransmitter regulating the release of another neurotransmitter) that modulate the membrane potential. While the multivariate regression analysis can identify a possible target that drives the clinical outcome (for instance the 5-HT_2_AR in the case of the unexpected high EPS liability of JNJ), the computer-based mechanistic mode can add the appeal of quantitatively understanding the neurobiology, i.e. clarifying the link from receptor modulation to membrane excitability through modulation of specific ion channels in specific parts of the neuronal network. In addition, in contrast to the mechanism-based computer model, multivariate regression analysis is unable to predict the outcome of a new target that hasn't been tested in the clinic before, or the effect of comedication often used in clinical trials.

The failure of the model to predict ocaperidone clinical outcome may be due to imperfect representation of the off-target physiology in the model. Alternatively, with steady-state dosing, the ocaperidone levels could accumulate leading to increased functional brain concentrations. Indeed hypothetical higher D_2_ receptor occupancy for ocaperidone substantially reduces the differences between predicted and reported values for EPS liability and PANSS total score. In that regard it is interest to note that ocaperidone is much more potent in vivo than haloperidol or risperidone with ED_50_ values in the amphetamine test below 1 microg/kg [58]. Additionally, for missing data we assumed the drug did not affect those receptors and that affinities to rodent receptors are identical for human receptors, but species differences in affinity are commonly present.

There are several issues, however, for which the model falls short. First, the results represent a relative difference from baseline, rather than an absolute predictor of clinical outcome. However, this approach is the only ‘preclinical’ model that predicts actual PANSS total score or EPS liability outcomes, in contrast to animal models that give more of a binary prediction.

The model fell short on the absolute prediction of the placebo improvement. The increased placebo improvement which has been observed lately in clinical trials cannot be effectively modeled by this approach, because they are presumably associated with issues like expectancy bias on the part of the investigator and the patient [59], [60]. Additionally, the model has been calibrated using historical values for the placebo effect collected in 26 different papers since 1988, where the placebo effect was much less prominent.

It is also important to realize that the model predictions are limited by the current state of knowledge. For example, the computer-based model is much less effective in predicting akathisia-related side-effects compared to Parkinsonian type side-effects [9]. Although historically it has been classified as an extrapyramidal disorder [61], akathisia might be driven by pathophysiological mechanisms more reflective of anxiety than motor signs [62]. The current version of the computer-based EPS model is focused on the cortico-nigrostriatal-thalamic pathway pathology and does not take into account other extrastriatal pathways.

The current EPS computer model is limited to Parkinsonian physiology and is well calibrated with historical data from patients initiated on anticholinergic medication to treat EPS symptoms. This might lead to differences between potential and expressed pathological changes - i.e., the drug may have increased EPS liability, but its expression in humans may not correspond to a given clinical readout unless very large numbers of patients are used. For instance, high EPS liability may not be optimally assessed by the use of anticholinergics, which is an ‘all-or-none’ approach that depends both on the subjects' description of the event and the physician's ability to elicit, characterize and manage that symptom. However the results suggest that the model correctly captures the ranking of the investigative drugs as compared to olanzapine with regard to the EPS liability. The platform also has reasonable correlations with some other measures of EPS liability [9], such as Simpson-Angus Scale (SAS) and the Abnormal Involuntary Movement Scale (AIMS) which capture different clinical aspects of this complex side-effect profile.

It is of interest to compare the predictivity of this computer-based modeling approach with the more traditional animal models currently used in psychiatry Research & Development. Both JNJ37822681 [41] and ocaperidone [46] passed all preclinical animal tests to the point that they were deemed of interest for a (financial and resource-intensive) investment in clinical development. Yet the computer model would have been able to raise a red flag about the EPS liability for JNJ37822681, because it quantitatively showed that the fast dissociating properties at the D2R did not compensate for the lack of effect at the cortical 5-HT_2_AR. The computer model further predicted a lack of clinically relevant differentiation between ocaperidone and olanzapine and suggested that higher doses of ocaperidone would reduce the therapeutic ratio between effect on PANSS Total and EPS liability. In addition, we are not aware of preclinical animal models that can quantitatively predict a PANSS total score, especially in comparison to an existing drug on the market.

The computer-based model has been calibrated using average values of treatment groups and do not reflect inter-individual differences caused by differences in individual genotypes and co-medications; however, the model, in principle can accommodate genotypic profiles if such information is obtained from the patient population evaluated, for instance through imaging genetics [63].

We chose to focus on the PANSS Total as readout because this is usually the primary readout for clinical trials with antipsychotics and there are more historical data available. We have been testing our computer model against other subscales, such as PANSS positive and PANSS negative subscales, for which we have less published data. Not unexpectedly, the calibration with PANSS positive subscale is very similar to the PANSS Total scale and the relative effect of the drugs on the PANSS positive scale is similar to their effect on PANSS Total.

Future developments include the implementation of more detailed subcortical anatomy and physiology [64] that will take into account the different properties of the direct versus indirect pathway in combination with detailed modeling of the globus pallidus interna and externa, the subthalamic nucleus and part of the thalamus. Alternatively other receptor types and neurotransmitter systems can be implemented in the appropriate brain region to build a model that is for instance, more suited for cognitive or negative symptoms Such an approach could, in principle, lead to other models for Parkinson's and Huntington's diseases.

Current preclinical animal models generally provide binary information relative to safety and efficacy, but they rarely predict relative performance of a novel investigative drug to a comparator. This computer-based mathematical model, calibrated retrospectively using published clinical data of many antipsychotic drugs can predict relative clinical outcomes, important in prioritizing discovery projects. In addition, when no target engagement data in humans are available, the computer-based model allows for the relative therapeutic window between PANSS effect and EPS liability to be estimated.

The ‘Quantitative Systems Pharmacology’ approach is being increasingly recognized as a possible translational tool for drug discovery and development in the field of oncology and metabolism [65] and contributed to a number of newly approved, rationally designed cancer drugs. Although the current understanding of human neurobiology in general and in schizophrenia pathology in particular is currently limited, the combination of the existing large academic expertise in computational neuroscience and the availability of endophenotype studies of the human brain using PET imaging and electroencephalogram (EEG) provides the framework for an increasingly more powerful ‘Quantitative Systems Pharmacology’ approach. In this context, it is of interest to note, that although the current version of the computer model is largely based upon existing dopamine dominated antipsychotic pharmacology; new cholinergic and glutamatergic targets can be readily introduced into the model based upon their preclinical physiology. As they in turn affect more complex neuronal network systems, like the type we model here; we expect that this ‘Quantitative Systems Pharmacology’ approach can yield better insights than pure qualitative reasoning as is done now.

In summary, although the current model did not perfectly predict the clinical outcome for the novel antipsychotic drugs, the comparative results against the active comparator were more reliable than could have been estimated by D_2_ binding properties or by preclinical animal model outcome. Further refinements using our expanded knowledge about receptor profiles and systems interaction should permit an even better predictive capacity. This approach can provide valuable insight into relative clinical efficacy and EPS liability, as well as into novel drug targets beyond the dopamine system and more efficiently drive drug development by enabling better selection of drugs prior to expensive and time-consuming clinical testing.

## References

[1] Lieberman JA (2007) Effectiveness of antipsychotic drugs in patients with chronic schizophrenia: efficacy, safety and cost outcomes of CATIE and other trials. J Clin Psychiatry 68 2: e04. 17335312 10.4088/jcp.0207e04

[2] Jaaro-Peled H, Ayhan Y, Pletnikov MV, Sawa A (2010) Review of pathological hallmarks of schizophrenia: comparison of genetic models with patients and nongenetic models. Schizophr Bull 36 2: 301–313. 19903746 10.1093/schbul/sbp133 PMC2833125

[3] Abi-Dargham A, Rodenhiser J, Printz D, Zea-Ponce Y, Gil R, et al (2000) Increased baseline occupancy of D_2_ receptors by dopamine in schizophrenia. Proc Natl Acad Sci U S A 97 14: 8104–8109. 10884434 10.1073/pnas.97.14.8104 PMC16677

[4] Meyer-Lindenberg A, Miletich RS, Kohn PD, Esposito G, Carson RE, et al (2002) Reduced prefrontal activity predicts exaggerated striatal dopaminergic function in schizophrenia. Nat Neurosci 5 3: 267–271. 11865311 10.1038/nn804

[5] Carlson CD, Cavazzoni PA, Berg PH, Wei H, Beasley CM, et al (2003) An integrated analysis of acute treatment-emergent extrapyramidal syndrome in patients with schizophrenia during olanzapine clinical trials: comparisons with placebo, haloperidol, risperidone, or clozapine. J Clin Psychiatry 64 8: 898–906. 12927004 10.4088/jcp.v64n0807

[6] Arvanitis LA, Miller BG (1997) Multiple fixed doses of “Seroquel” (quetiapine) in patients with acute exacerbation of schizophrenia: a comparison with haloperidol and placebo. The Seroquel Trial 13 Study Group. Biol Psychiatry 42 4: 233–246. 9270900 10.1016/s0006-3223(97)00190-x

[7] Advokat C, Dixon D, Schneider J, Comaty JE Jr (2004) Comparison of risperidone and olanzapine as used under “real-world” conditions in a state psychiatric hospital. Prog Neuropsychopharmacol Biol Psychiatry 28 3: 487–495. 15093956 10.1016/j.pnpbp.2003.11.015

[8] Copolov DL, Link CG, Kowalcyk B (2000) A multicentre, double-blind, randomized comparison of quetiapine (ICI 204,636, ‘Seroquel’) and haloperidol in schizophrenia. Psychol Med 30 1: 95–105. 10722180 10.1017/s0033291799001476

[9] Spiros A, Roberts P, Geerts H (2012) A quantitative systems pharmacology computer model for schizophrenia efficacy and extrapyramidal side-effects. Drug Develop Res 73 4: 1098–1109.

[10] Spiros A, Carr R, Geerts H (2010) Not all partial dopamine D(2) receptor agonists are the same in treating schizophrenia. Exploring the effects of bifeprunox and aripiprazole using a computer model of a primate striatal dopaminergic synapse. Neuropsychiatr Dis Treat 6: 589–603. 20856922 10.2147/NDT.S12460 PMC2938308

[11] Lisman JE, Coyle JT, Green RW, Javitt DC, Benes FM, et al (2008) Circuit-based framework for understanding neurotransmitter and risk gene interactions in schizophrenia. Trends Neurosci 31 5: 234–242. 18395805 10.1016/j.tins.2008.02.005 PMC2680493

[12] Grace AA (2000) Gating of information flow within the limbic system and the pathophysiology of schizophrenia. Brain Res Brain Res Rev 31 2–3: 330–341. 10719160 10.1016/s0165-0173(99)00049-1

[13] Hines ML, Carnevale NT (1997) The NEURON simulation environment. Neural Comput 9 6: 1179–1209. 9248061 10.1162/neco.1997.9.6.1179

[14] Haber SN, Rauch SL (2010) Neurocircuitry: a window into the networks underlying neuropsychiatric disease. Neuropsychopharmacology 35 1: 1–3. 20010702 10.1038/npp.2009.146 PMC3055437

[15] Abdallah L, Bonasera SJ, Hopf FW, O'Dell L, Giorgetti M, et al (2009) Impact of serotonin 2C receptor null mutation on physiology and behavior associated with nigrostriatal dopamine pathway function. J Neurosci 29 25: 8156–8165. 19553455 10.1523/JNEUROSCI.3905-08.2009 PMC3077993

[16] Porras G, De Deurwaerdere P, Moison D, Spampinato U (2003) Conditional involvement of striatal serotonin3 receptors in the control of in vivo dopamine outflow in the rat striatum. Eur J Neurosci 17 4: 771–781. 12603267 10.1046/j.1460-9568.2003.02512.x

[17] Perez-Rosello T, Figueroa A, Salgado H, Vilchis C, Tecuapetla F, et al (2005) Cholinergic control of firing pattern and neurotransmission in rat neostriatal projection neurons: role of CaV2.1 and CaV2.2 Ca2+ channels. J Neurophysiol 93 5: 2507–2519. 15615835 10.1152/jn.00853.2004

[18] Shen W, Hamilton SE, Nathanson NM, Surmeier DJ (2005) Cholinergic suppression of KCNQ channel currents enhances excitability of striatal medium spiny neurons. J Neurosci 25 32: 7449–7458. 16093396 10.1523/JNEUROSCI.1381-05.2005 PMC6725301

[19] Hersch SM, Gutekunst CA, Rees HD, Heilman CJ, Levey AI (1994) Distribution of m1-m4 muscarinic receptor proteins in the rat striatum: light and electron microscopic immunocytochemistry using subtype-specific antibodies. J Neurosci 14 5 Pt 2: 3351–3363. 8182478 10.1523/JNEUROSCI.14-05-03351.1994 PMC6577507

[20] Braga MF, Aroniadou-Anderjaska V, Manion ST, Hough CJ, Li H (2004) Stress impairs alpha(1A) adrenoceptor-mediated noradrenergic facilitation of GABAergic transmission in the basolateral amygdala. Neuropsychopharmacology 29 1: 45–58. 14532911 10.1038/sj.npp.1300297

[21] Falk T, Xie JY, Zhang S, Kennedy J, Bennett J, et al (2008) Over-expression of the potassium channel Kir2.3 using the dopamine-1 receptor promoter selectively inhibits striatal neurons. Neuroscience 155 1: 114–27. 18571331 10.1016/j.neuroscience.2008.04.075

[22] Kuzhikandathil EV, Oxford GS (2002) Classic D1 dopamine receptor antagonist R-(+)-7-chloro-8-hydroxy-3-methyl-1-phenyl-2,3,4,5-tetrahydro-1H-3-benzaze pine hydrochloride (SCH23390) directly inhibits G protein-coupled inwardly rectifying potassium channels. Mol Pharmacol 62 1: 119–26. 12065762 10.1124/mol.62.1.119

[23] Gruber AJ, Solla SA, Surmeier DJ, Houk JC (2003) Modulation of striatal single units by expected reward: a spiny neuron model displaying dopamine-induced bistability. J Neurophysiol 90 2: 1095–114. 12649314 10.1152/jn.00618.2002

[24] Mermelstein PG, Song WJ, Tkatch T, Yan Z, Surmeier DJ (1998) Inwardly rectifying potassium (IRK) currents are correlated with IRK subunit expression in rat nucleus accumbens medium spiny neurons. J Neurosci 18 17: 6650–61. 9712637 10.1523/JNEUROSCI.18-17-06650.1998 PMC6792959

[25] Hines ML, Carnevale NT (1997) The NEURON simulation environment. Neural Comput 9 6: 1179–209. 9248061 10.1162/neco.1997.9.6.1179

[26] Bamford NS, Robinson S, Palmiter RD, Joyce JA, Moore C, et al (2004) Dopamine modulates release from corticostriatal terminals. J Neurosci 24 43: 9541–52. 15509741 10.1523/JNEUROSCI.2891-04.2004 PMC6730145

[27] Gurevich EV, Bordelon Y, Shapiro RM, Arnold SE, Gur RE, et al (1997) Mesolimbic dopamine D3 receptors and use of antipsychotics in patients with schizophrenia. A postmortem study. Arch Gen Psychiatry 54 3: 225–232. 9075463 10.1001/archpsyc.1997.01830150047009

[28] Herrero MT, Barcia C, Navarro JM (2002) Functional anatomy of thalamus and basal ganglia. Childs Nerv Syst 18 8: 386–404. 12192499 10.1007/s00381-002-0604-1

[29] Marek GJ, Wright RA, Gewirtz JC, Schoepp DD (2001) A major role for thalamocortical afferents in serotonergic hallucinogen receptor function in the rat neocortex. Neuroscience 105 2: 379–92. 11672605 10.1016/s0306-4522(01)00199-3

[30] Aznar S, Qian Z, Shah R, Rahbek B, Knudsen GM (2003) The 5-HT1A serotonin receptor is located on calbindin- and parvalbumin-containing neurons in the rat brain. Brain Res 959 1: 58–67. 12480158 10.1016/s0006-8993(02)03727-7

[31] Gradinaru V, Mogri M, Thompson KR, Henderson JM, Deisseroth K (2009) Optical deconstruction of parkinsonian neural circuitry. Science 324 5925: 354–9. 19299587 10.1126/science.1167093 PMC6744370

[32] Carr DB, Cooper DC, Ulrich SL, Spruston N, Surmeier DJ (2002) Serotonin receptor activation inhibits sodium current and dendritic excitability in prefrontal cortex via a protein kinase C-dependent mechanism. J Neurosci 22 16: 6846–55. 12177182 10.1523/JNEUROSCI.22-16-06846.2002 PMC6757866

[33] Foehring RC (1996) Serotonin modulates N- and P-type calcium currents in neocortical pyramidal neurons via a membrane-delimited pathway. J Neurophysiol 75 2: 648–59. 8714642 10.1152/jn.1996.75.2.648

[34] Brown P, Mazzone P, Oliviero A, Altibrandi MG, Pilato F, et al (2004) Effects of stimulation of the subthalamic area on oscillatory pallidal activity in Parkinson's disease. Exp Neurol 188 2: 480–90. 15246847 10.1016/j.expneurol.2004.05.009

[35] Magnin M, Morel A, Jeanmonod D (2000) Single-unit analysis of the pallidum, thalamus and subthalamic nucleus in parkinsonian patients. Neuroscience 96 3: 549–64. 10717435 10.1016/s0306-4522(99)00583-7

[36] Koh PO, Bergson C, Undie AS, Goldman-Rakic PS, Lidow MS (2003) Up-regulation of the D1 dopamine receptor-interacting protein, calcyon, in patients with schizophrenia. Arch Gen Psychiatry 60 3: 311–9. 12622665 10.1001/archpsyc.60.3.311

[37] Silvestri S, Seeman MV, Negrete JC, Houle S, Shammi CM, et al (2000) Increased dopamine D2 receptor binding after long-term treatment with antipsychotics in humans: a clinical PET study. Psychopharmacology (Berl) 152 2: 174–80. 11057521 10.1007/s002130000532

[38] Laakso A, Bergman J, Haaparanta M, Vilkman H, Solin O, et al (2001) Decreased striatal dopamine transporter binding in vivo in chronic schizophrenia. Schizophr Res 52 1–2: 115–20. 11595398 10.1016/s0920-9964(00)00095-5

[39] Van Oekelen D, Luyten WH, Leysen JE (2003) Ten years of antisense inhibition of brain G-protein-coupled receptor function. Brain Res Brain Res Rev 42 2: 123–42. 12738054 10.1016/s0165-0173(03)00153-x

[40] Scarr E, Keriakous D, Crossland N, Dean B (2006) No change in cortical muscarinic M2, M3 receptors or [35S]GTPgammaS binding in schizophrenia. Life Sci 78 11: 1231–7. 16214178 10.1016/j.lfs.2005.06.038

[41] Langlois X, Lavreysen H, Atack J, Cik M, Macdonald G, et al (2012) Pharmacology of JNJ-37822681, a specific and fast-dissociating D2 antagonist for the treatment of schizophrenia. J Pharmacol Exp Ther 342 1: 91–105. 22490380 10.1124/jpet.111.190702

[42] Schmidt ME, Kent JM, Daly E, Janssens L, Van Osselaer N, et al (2012) A double-blind, randomized, placebo-controlled study with JNJ-37822681, a novel, highly selective, fast dissociating D92) receptor antagonist in the treatment of acute exacerbation of schizophrenia. Eur Neuropsycholopharmacol 30 March [epub ahead of print]. 10.1016/j.euroneuro.2012.02.007 22464973

[43] Seeman P, Tallerico T (1999) Rapid release of antipsychotic drugs from dopamine D2 receptors: an explanation for low receptor occupancy and early clinical relapse upon withdrawal of clozapine or quetiapine. Am J Psychiatry 156 6: 876–884. 10360126 10.1176/ajp.156.6.876

[44] Seeman P (2002) Atypical antipsychotics: mechanism of action. Can J Psychiatry 47 1: 27–38. 11873706

[45] Tresadern G, Bartolome JM, Macdonald GJ, Langlois X (2011) Molecular properties affecting fast dissociation from the D2 receptor. Bioorg Med Chem 19 7: 2231–2241. 21421319 10.1016/j.bmc.2011.02.033

[46] Leysen JE, Janssen PM, Gommeren W, Wynants J, Pauwels PJ, et al (1992) In vitro and in vivo receptor binding and effects on monoamine turnover in rat brain regions of the novel antipsychotics risperidone and ocaperidone. Mol Pharmacol 41 3: 494–508. 1372084

[47] Agid O, Kapur S, Arenovich T, Zipursky RB (2003) Delayed-onset hypothesis of antipsychotic action: a hypothesis tested and rejected. Arch Gen Psychiatry 60 12: 1228–1235. 14662555 10.1001/archpsyc.60.12.1228

[48] Talvik M, Nordstrom AL, Okubo Y, Olsson H, Borg J, et al (2006) Dopamine D2 receptor binding in drug-naive patients with schizophrenia examined with raclopride-C11 and positron emission tomography. Psychiatry Res 148 2–3: 165–173. 17095199 10.1016/j.pscychresns.2006.05.009

[49] Geddes J, Freemantle N, Harrison P, Bebbington P (2000) Atypical antipsychotics in the treatment of schizophrenia: systematic overview and meta-regression analysis. Br Med J 321 7273: 1371–1376. 11099280 10.1136/bmj.321.7273.1371 PMC27538

[50] Davis JM, Chen N, Glick ID (2003) A meta-analysis of the efficacy of second-generation antipsychotics. Arch Gen Psychiatry 60 6: 553–564. 12796218 10.1001/archpsyc.60.6.553

[51] Kapur S, Zipursky RB, Remington G, Jones C, DaSilva J, et al (1998) 5-HT2 and D2 receptor occupancy of olanzapine in schizophrenia: a PET investigation. Am J Psychiatry 155 7: –928. 10.1176/ajp.155.7.921 9659858

[52] Nyberg S, Dencker SJ, Malm U, Dahl ML, Svenson JO, et al (1998) D(2)- and 5-HT(2) receptor occupancy in high-dose neuroleptic-treated patients. Int J Neuropsychopharmacol 1 2: 95–101. 11281952 10.1017/S1461145798001229

[53] Samtani MN, Gopal S, Gassmann-Mayer C, Alphs L, Palumbo JM (2011) Dosing and switching strategies for paliperidone palmitate: based on population pharmacokinetic modelling and clinical trial data. CNS Drugs 25 10: 829–845. 21936586 10.2165/11591690-000000000-00000

[54] Nordstrom AL, Farde L, Wiesel FA, Forslund K, Pauli S, et al (1993) Central D2-dopamine receptor occupancy in relation to antipsychotic drug effects: a double-blind PET study of schizophrenic patients. Biol Psychiatry 33 4: 227–235. 8097114 10.1016/0006-3223(93)90288-o

[55] Geerts H (2009) Of mice and men: bridging the translational disconnect in CNS drug discovery. CNS Drugs 23 11: 915–926. 19845413 10.2165/11310890-000000000-00000

[56] Werneck AL, Rosso AL, Vincent MB (2009) The use of an antagonist 5-HT2a/c for depression and motor function in Parkinson' disease. Arq Neuropsiquiatr 67 2B: 407–412. 19623435 10.1590/s0004-282x2009000300007

[57] Andersen J, Korner A, Ostergaard P, Fensbo C, Birket-Smith M, et al (1990) A double blind comparative multicentre study of remoxipride and haloperidol in schizophrenia. Acta Psychiatr Scand Suppl 358: 104–107. 10.1111/j.1600-0447.1990.tb05299.x 1978467

[58] Megens AA, Awouters FH, Meert TF, Schellekens KH, Niemegeers CJ, et al (1992) Pharmacological profile of the new potent neuroleptic ocaperidone (R 79,598). J Pharmacol Exp Ther 260 1: 146–59. 1370538

[59] Kemp AS, Schooler NR, Kalali AH, Alphs L, Anand R, et al (2010) What is causing the reduced drug-placebo difference in recent schizophrenia clinical trials and what can be done about it? Schizophr Bull 36 3: 504–509. 18723840 10.1093/schbul/sbn110 PMC2879679

[60] Alphs L, Benedetti F, Fleischhacker WW, Kane JM (2012) Placebo-related effects in clinical trials in schizophrenia: what is driving this phenomenon and what can be done to minimize it? Int J Neuropsychopharmacol 1–12. 10.1017/S1461145711001738 PMC3405769 22217384

[61] Dayalu P, Chou KL (2008) Antipsychotic-induced extrapyramidal symptoms and their management. Expert Opin Pharmacother 9 9: 1451–1462. 18518777 10.1517/14656566.9.9.1451

[62] Sachdev P, Kruk J (1994) Clinical characteristics and predisposing factors in acute drug-induced akathisia. Arch Gen Psychiatry 51 12: 963–974. 7979885 10.1001/archpsyc.1994.03950120035007

[63] Turner JA, Smyth P, Macciardi F, Fallon JH, Kennedy JL, et al (2006) Imaging phenotypes and genotypes in schizophrenia. Neuroinformatics 4 1: 21–49. 16595857 10.1385/NI:4:1:21

[64] Terman D, Rubin JE, Yew AC, Wilson CJ (2002) Activity patterns in a model for the subthalamopallidal network of the basal ganglia . J Neurosci 22 7: 2963–2976. 11923461 10.1523/JNEUROSCI.22-07-02963.2002 PMC6758326

[65] Sorger PK, Allerheiligen SRB, Abernethy DR, Altman RB, Brouwer KLR, et al. (2011) Quantitative and systems pharmacology in the post-genomic era: New approaches to discovering drugs and understanding therapeutic mechanisms. Available: http://www.scribd.com/doc/71263148/systems. Accessed 9 May 2012.

